# Latest methods of fluorescence-based protein crystal identification

**DOI:** 10.1107/S2053230X15000114

**Published:** 2015-01-28

**Authors:** Arne Meyer, Christian Betzel, Marc Pusey

**Affiliations:** a Laboratory for Structural Biology of Infection and Inflammation, University of Hamburg, c/o DESY Building 22a, Notkestrasse 85, 22607 Hamburg, Germany; b iXpressGenes Inc., 601 Genome Way, Huntsville, AL 35806, USA

**Keywords:** protein crystal identification, fluorescence

## Abstract

Successful protein crystallization screening experiments are dependent upon the experimenter being able to identify positive outcomes. The introduction of fluorescence techniques has brought a powerful and versatile tool to the aid of the crystal grower. Trace fluorescent labeling, in which a fluorescent probe is covalently bound to a subpopulation (<0.5%) of the protein, enables the use of visible fluorescence. Alternatively, one can avoid covalent modification and use UV fluorescence, exploiting the intrinsic fluorescent amino acids present in most proteins. By the use of these techniques, crystals that had previously been obscured in the crystallization drop can readily be identified and distinguished from amorphous precipitate or salt crystals. Additionally, lead conditions that may not have been obvious as such under white-light illumination can be identified. In all cases review of the screening plate is considerably accelerated, as the eye can quickly note objects of increased intensity.

## Abstract 2

Fluorescence, whether intrinsic or by using trace fluorescent labeling, can be a powerful aid in macromolecule crystallization. Its use in screening for crystals is discussed here.

## 1. Introduction   

The purpose of screening experiments is the determination of the main factors that lead to successful crystallization of the system under investigation. Once identified, subsequent optimization tests are carried out to improve the effects of the main factors (Mason *et al.*, 2003 ▶; Myers *et al.*, 2009 ▶). A consequence is that the screening is often expanded to many hundreds or even thousands of conditions to maximize combinatorial chemical space coverage and thus the chances of a successful (crystalline) outcome. This reduces screening trial volumes to enable additional experiments and promotes the use of automation to set up and then monitor the experiments. Owing to the cost of the latter, many smaller sized structure groups find the ‘survey everything’ approach out of reach.

Macromolecule crystallization screening is at present typically carried out as a binary process, that is, no (0) there is not a crystal in this well or yes (1) there is a crystal. Unrecognized by the experimenter, and usually unrealized, are the biophysical events that went into the observed outcome. Although in general conceptually understood, the realisation that crystal nucleation is a stochastic process is not factored into the screening process or the results observed. This last point is usually taken into account during subsequent optimization trials, where replicates are often set up. However, the replicates are often composed more to provide additional crystals than to pay homage to random chance. As an end result, many crystallization screening experiments with potentially positive outcomes are missed, leading to the setup of a large number of different screens in an effort to capture that elusive positive outcome. It has been calculated that only 300 conditions would be sufficient to successfully crystallize a protein possessing a crystallization chance of 2% (Segelke, 2001 ▶). However, this assessment assumes that every positive condition will invariably produce a crystal, ignoring the stochastic nature of the nucleation process.

Whether manually scanning through crystallization screening plates, or viewing hundreds of images obtained by an automated imaging system, the review process can quickly become a mind-numbing task. Close examination of each drop must be carried out, with the experimenter constantly addressing the question of whether the observed features are macromolecule crystals or not. Using white light this distinction can be suggested by the presence of objects with edges or straight lines, but again one can be confounded when these objects prove to be salt, not protein, crystals. Intensity is typically a more rapid search parameter than geometry, and this problem becomes greatly simplified by using fluorescence, where only the protein is visible.

When a crystallization screening experiment is carried out we apply an extensive range of chemical environments to the target protein. The observed outcomes are the results of the interactions of the protein with these test conditions. Practical experience tells us that these outcomes vary, and that the variations observed must be a function of the conditions imposed. The particular methods employed for the screening experiment (vapor diffusion, batch *etc*.) can also provide insight into the temporal characteristics of this response. The observable results with the individual wells can also provide additional information about the screening experiment. Fluorescence is becoming an increasingly popular method for observing crystallization wells; here, we review fluorescence-based approaches to obtaining useful screening information, with emphasis on the UV system of XtalConcepts and the visible-light method of iXpressGenes/Molecular Dynamics.

## 2. Procedures and instrumentation   

Intrinsic fluorescence imaging of proteins rests predominantly on the fluorescence properties of tryptophan. For the examples reported here, imaging by use of native fluorescence was carried out using the XtalLight 100 (XtalConcepts GmbH, Germany). The instrument uses a mercury-vapor UV source coupled to a quartz light guide with an inclined beam, in a near-epifluorescence arrangement, for the excitation that bypasses the microscope optics, with a 400 nm UV filter (Rapp Optoelectronic, Hamburg, Germany) to remove the source illumination from the emission path (Dierks *et al.*, 2010 ▶).

Visible fluorescent imaging of screening plates is carried out using a Crystal X2 imaging system (iXpressGenes/Molecular Dynamics). The instrument is equipped with two epifluorescent (produced by reflected rather than transmitted light) sources and one white-light (transmission) source. Plate positioning, source illumination and image capture and storage are all controlled through a PC.

Trace fluorescent labeling (TFL) of proteins using succinimidyl ester-activated fluorescent probes was carried out as described elsewhere (Pusey *et al.*, submitted). The procedure is derived from previously published methods (Forsythe *et al.*, 2006 ▶; Pusey *et al.*, 2007 ▶). The given procedure is for labeling random side-chain amines. The N-terminal amine, sulfhydryl and other reactive sites of a protein may be specifically targeted through the selection of some combination of the fluorescent probe and the labeling conditions.

## 3. Fluorescence methods   

Fluorescence occurs when an electron of a molecule is excited to a higher quantum state and then relaxes to the ground state with the emission of a photon. When stimulation is by light the emitted light is typically at a lower energy (a higher wavelength), with the emission intensity proportional to that of the excitation intensity. There are several important characteristics of fluorescence that need to be borne in mind when setting up to make measurements. Firstly, there is the Stokes shift, the difference between the maximum wavelengths of excitation and emission. This determines how well one can remove the excitation from the emission signal. Fluors may have more than one excitation or emission peak, although many of the properties that are experimentally exploited can vary with the wavelength for any given probe. Owing to the absorption spectral range of typical fluors, one can use an excitation wavelength that is well removed from the excitation maximum to overcome problems with a small Stokes shift, but this will be at the expense of the emission signal intensity. The absorptivity and quantum-yield characteristics of the fluorescing species are important. The absorptivity is an indication of how well the molecule absorbs the excitation light, while the quantum yield is a measure of the efficiency by which the molecule returns a photon for every photon absorbed. The absorptivity is determined at a specific wavelength, while the quantum yield is determined over the emission spectrum of the molecule.

There are three readily accessible approaches extant to using fluorescence as an aid in protein crystallization screening. Firstly, the naturally occurring and well known fluorescence properties of the constituent amino acids, most notably tryptophan, can be used, as described by Judge *et al.* (2005 ▶). Asanov *et al.* (2001 ▶) further proposed the use of intrinsic fluorescence as a means of assessing protein crystal diffraction quality. Secondly, a noncovalent dye can be used to indicate crystals (Groves *et al.*, 2007 ▶). Thirdly, trace levels of covalently bound dye can be used to identify crystals (Forsythe *et al.*, 2006 ▶). A starting advantage of all three methods is that salt crystals, a bane of macromolecular crystallographers, do not show up, and are thus readily removed from consideration. A second advantage, for fluorescence in general, is that in a well designed system the excitation light is removed from the observed signal, and thus one only obtains signal from the fluorescing species, enhancing the ability to detect it. For all three methods this is designed to be just the protein. Thirdly, the fluorescence intensity is proportional to that of the intensity of the excitation light and the concentration of the fluorescing species. Crystals are typically the most densely packed form for proteins, and thus should be the brightest objects visible in the field, leading to the expectation in these experiments that intensity is proportional to structure. For all three methods this simplifies observations to a search for emission-intense objects. The differences between the methods are primarily in their ease of implementation and in their usage.

The use of naturally occurring fluorescent amino acids for crystallization screening, as described by Judge *et al.* (2005 ▶), has since been incorporated into several commercially available crystallization-plate imaging systems. The major advantage of this approach is in its ease of use: just set up the plate, with no additional pre-handling or post-handling of the protein necessary. However, all materials and optics in the path of the excitation illumination should be relatively UV-transparent, which can add considerably to the cost. Background fluorescence is present from some crystallization cocktail components, notably the PEG MMEs (Judge *et al.*, 2005 ▶), but this was found to be less than 30% of that of proteins. Intrinsic protein fluorescence is highly variable, dependent upon the number of tryptophans present, and even more so when one considers that the local environment, that is, the crystallization conditions, may affect fluorescence. To some extent environmental effects are likely to be ameliorated by the burying of the fluorescent species within the crystal, giving a double benefit in the reduction of quenching and signal increase by concentration.

The work of Judge *et al.* (2005 ▶) also shows how to and how not to set up a fluorescence microscopy system. Their Fig. 2 shows a ‘proper’ epifluorescence microscopy system, in which the excitation-light direction is 180° from that of the emission light, effectively removing much of it from the signal. Their Fig. 3 shows a layout in which the excitation and signal light directions are the same, in which the excitation shines directly into the detector. This layout removes one of the benefits of fluorescence, the removal of the source illumination from the signal, as illustrated in their Fig. 7. While the fluorescent crystals are visible, the background is also very bright, reducing the contrast.

**Figure 1:**
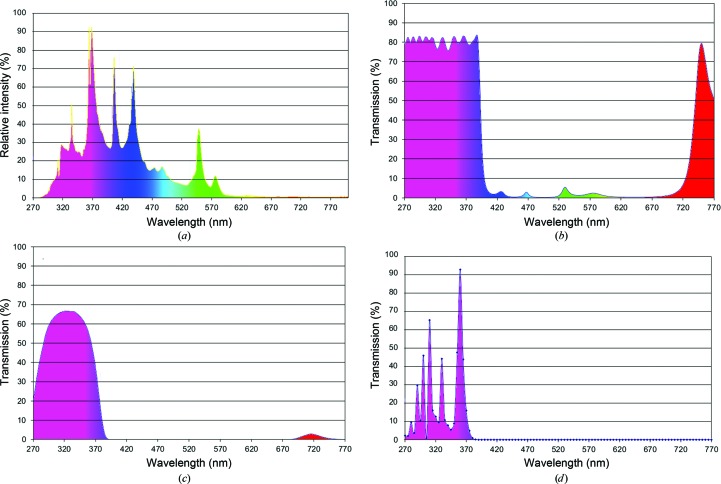
(*a*) The mercury vapor lamp spectrum of the light source, XtalLight 100, used for the experiments (source spectrum). (*b*) Transmission spectrum of the 385 nm short-pass interference filter applied to remove visible light from the source spectrum. (*c*) Transmission spectrum of the UV glass absorption filter applied to remove remaining visible light, especially of wavelengths above 670 nm which can permit the short-pass 385 nm filter. (*d*) Calculated spectrum of the light source after filtering the so-called illumination spectrum. The illumination spectrum has been calculated by multiplication of the unfiltered mercury arc lamp emission spectrum with the transmission spectra of the UV-transparent glass filter (UG-filter) and the the short-pass 385 nm filter and the permeability of glass cover slips.

An alternative approach to implementing intrinsic UV fluorescence imaging has been introduced by XtalConcepts with their XtalLight 100 system. This uses a mercury short-arc lamp for the excitation source, which is directed to the sample by a quartz fiber-optic light guide (Dierks *et al.*, 2010 ▶). A typical source spectrum is shown in Fig. 1 ▶(*a*). The illumination spectrum is generated when the source spectrum is filtered by a short-pass 385 nm filter (Fig. 1 ▶ *b*) and a visible-light absorption filter (UG-filter; Fig. 1 ▶ *c*) to remove excitation light in the detectable tryptophan fluorescence emission range. Finally, the filtered spectrum was multiplied by the transmission curve of 0.16 mm thick glass cover slides. The resulting excitation spectrum is shown in Fig. 1 ▶(*d*). The most prominent available excitation peaks of the lamp’s spectrum are at wavelengths above 300 nm. On the imaging side, the only required modification in the optical path is a long-pass 400 nm UV filter (Rapp Optoelectronic, Hamburg, Germany). This filter is required because most CCD chips are sensitive to UV light and, depending on the glass, the thickness of the glass lenses in the optical pathway is not sufficient to completely absorb the source UV light. This overall approach allows the use of protein intrinsic fluorescence without having to resort to higher cost quartz optics or UV-transmissive screening plates or covers.

**Figure 2:**
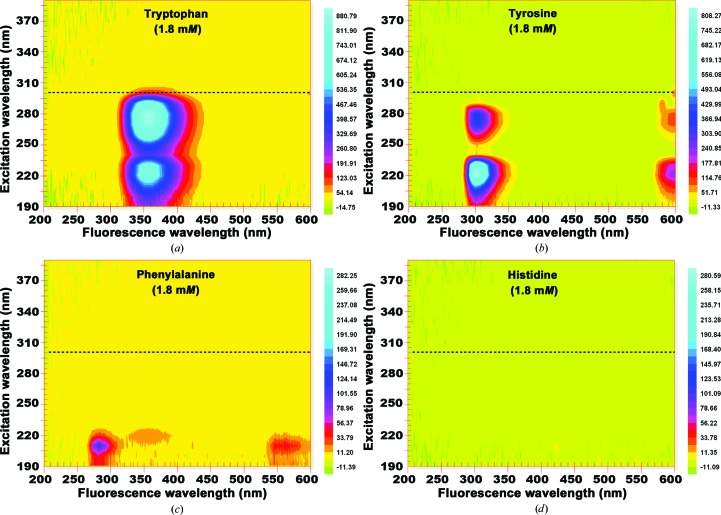
Fluorescence spectra in the context of the applied excitation spectra of the four aromatic amino acids tryptophan (*a*), tyrosine (*b*), phenylalanine (*c*) and histidine (*d*) in water at 1.8 m*M*. The dashed line indicates an excitation wavelength of 300 nm. For intrinsic fluorescence only tryptophan is relevant. Tyrosine fluorescence excitation is irrelevant for intrinsic fluorescence imaging owing to its weak absorption when illuminated with the applied illumination spectrum.

It has long been known that proteins contain four aromatic amino-acid residues (tryptophan, tyrosine, phenylalanine and histidine) which may contribute to the intrinsic fluorescence of a protein (Asanov *et al.*, 2001 ▶). In contrast to fluorescence imaging of proteins based on trace fluorescence labeling, intrinsic fluorescence imaging rests predominantly on the fluorescence properties of tryptophan. Tryptophan has a fluorescence excitation maximum at a wavelength of 280 nm with maximum emitted fluorescence light at 350 nm (Teale & Weber, 1957 ▶; Fig. 2 ▶ *a*) depending on the polarity of its close environment. Most plate-covering materials have a low opacity for UV wavelengths, especially the glass used for cover slips, glass cover plates and capillaries. An effective excitation spectrum for intrinsic fluorescence imaging as a detection tool is therefore expected to be in the range of 280 nm to approximately 300 nm, owing to the emission spectra of the available UV-light sources. Interestingly, for intrinsic fluorescence imaging of protein crystals, another part of the spectrum has been determined to be very useful for protein crystals enclosed in crystallization containers (Dierks *et al.*, 2010 ▶).

Technically, intrinsic fluorescence imaging is easily adaptable to existing microscopes and imaging systems, as UV light can easily be transferred by quartz-fiber light guides. For example, the UV-light source used here (XtalLight 100, XtalConcepts GmbH, Germany) is adaptable to most of the available commercial imaging systems or conventional light microscopes that are equipped with a CCD camera. Intrinsic fluorescence imaging can be applied to macromolecules other than proteins, even when they possess weak intrinsic fluorescence owing to the absence of fluorophores. Specific fluorescence dyes are also available that allow extended intrinsic fluorescence imaging, for example for nucleic acids using the SYBR Gold fluorescence staining dye (Uyeno *et al.*, 2004 ▶). This dye binds specifically but not covalently to nucleic acids and provides the polymer with sufficient fluorescence property to apply intrinsic fluorescence imaging.

**Figure 3:**
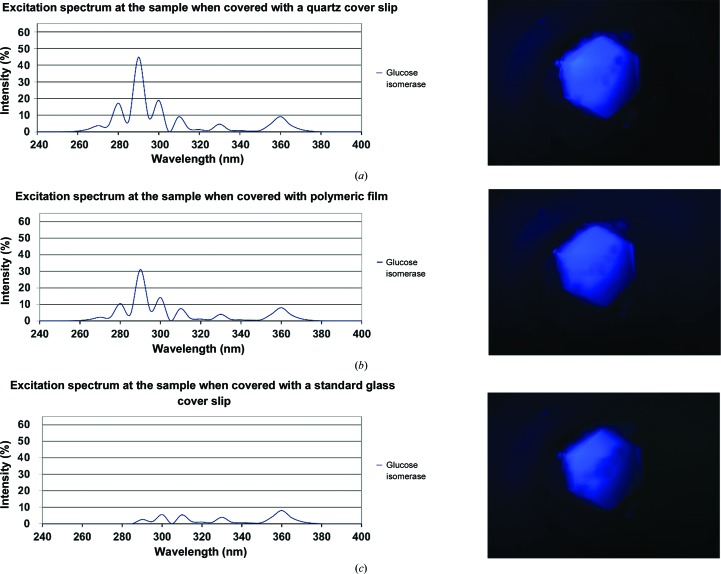
The diagrams on the left show the calculated excitation spectra at the sample for three different cover materials. On the right, intrinsic fluorescence images of the same glucose isomerase crystal covered with these three covering materials are shown. To allow a 1:1 comparison, the exposure time and all other camera parameters were kept identical for all three cases. Although the excitation spectra at short wavelengths (>300 nm) are vastly different owing to the absorption properties of the cover material, the intrinsic fluorescence image is still remarkably clear. The fact that even longer wavelengths are sufficiently effective to excite intrinsic fluorescence could be explained by tryptophan fluorescence properties, namely ineffective at wavelength larger than 300 nm but overcompensated by (*a*) the strong emission of the light source at these wavelengths and (*b*) the high permeability of the covering materials for this regime of the spectrum.

Evaluating intrinsic fluorescence as a tool for laboratory applications, the fluorescence excitation efficiency has been investigated using crystals of biological macromolecules. The fluorescence excitation efficiency of the available source was analyzed by recording the spectral characteristics of the four aromatic amino acids (Fig. 2 ▶). The transmission spectra of covering materials regarding their UV-light opacity was determined using a spectrophotometer (Varian, Palo Alto, California, USA) with a quartz cuvette (Hellma fluorescence cell; catalog No. 105-252-85-40, Hellma, Germany). To evaluate the practical use of intrinsic fluorescence, images were taken of a crystal of glucose isomerase in a low-fluorescing crystallization plate (MRC-MAXI 48-well Crystallization Plate UV; catalog No. MD11-004U-100, Molecular Dimensions, UK). For each image of the crystal the covering material was exchanged, starting without any covering material, then a transparent film (ClearVue Sheets; catalog No. MD6-01S, Molecular Dimensions, UK) and finally a glass cover slip (catalog No. 01 010 40; Marienfeld, Germany), representing the two most common classes of well-sealing materials (Fig. 3 ▶). To allow direct comparison of the resulting images, the camera exposure time and gain value were kept constant. The use of polymeric film as a covering material (Fig. 3 ▶ *b*) reduces the intensity of wavelengths of <300 nm; however, the intrinsic fluorescence is almost as intensive as in Fig. 3 ▶(*a*). Even with glass, known to be a highly UV-absorbing material, as a cover (Fig. 3 ▶ *c*), intrinsic fluorescence remains clearly detectable. The reason for the remarkable efficiency of intrinsic fluorescence excitation is a combination of the spectral characteristics of the high-intensity light source (Fig. 1 ▶) with the excitation properties of protein tryptophan at wavelengths above 300 nm (Fig. 2 ▶), even when the intensity of wavelengths of <300 nm is significantly reduced.

**Figure 4:**
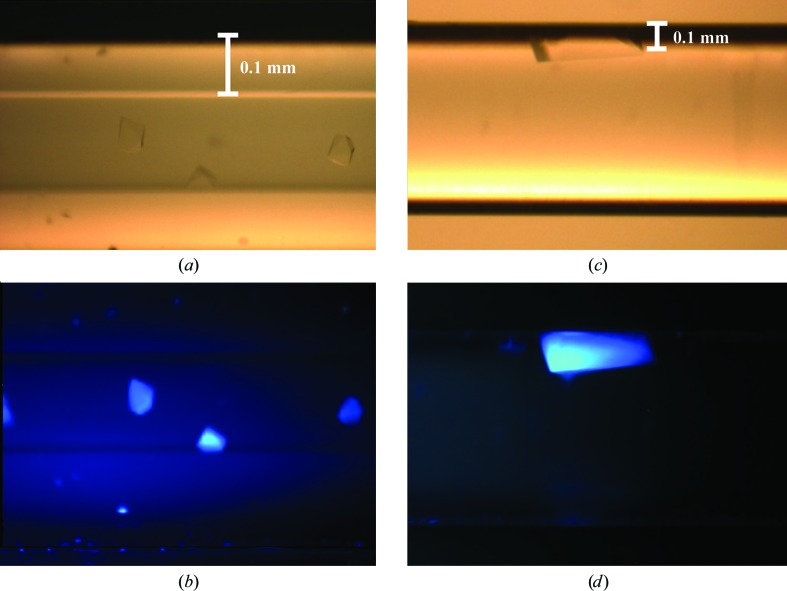
(*a*) Crystals of mistletoe lectin I inside a thick-walled glass capillary from a Gel Tube R (GT-R) crystallization kit used on JAXA-GCF flight No. 6 illuminated with white light. The crystal size is estimated to be 0.3 mm. (*b*) The same sample illuminated with UV light according to the excitation spectrum (Fig. 3 ▶). (*c*) Crystal of glucose isomerase obtained from a counter-diffusion crystallization experiment applying the GCB-2 Box (Triana). (*d*) The same crystal illuminated with UV light as in (*b*).

**Figure 5:**
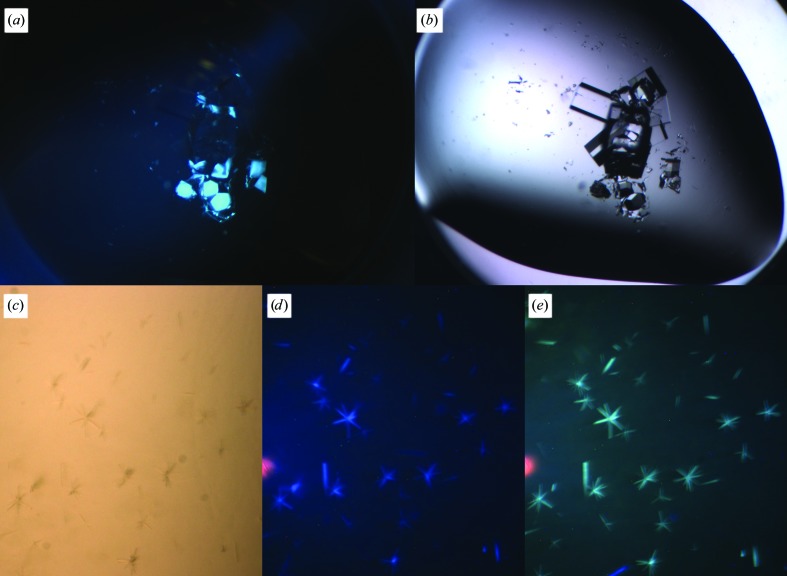
(*a*, *b*) Hen egg-white lysozyme crystals among sodium chloride crystals illuminated with filtered radiation from a mercury vapor lamp (*a*) and white light (*b*). The protein crystals show the typical intrinsic blue fluorescence of tryptophan, while the salt crystals are invisible when excited with this spectrum. (*c*) Crystals of the 40-mer RNA l-oligonucleotide in complex with the protein l-CCP2 illuminated with bright light. (*d*) The same crystals when illuminated with the excitation spectrum; crystals of the protein–RNA complex crystals show the intrinsic blue fluorescence of tryptophan. (*e*) The same crystals after the addition of SYBR Gold fluorescence stain (∼1:1000); the crystals show characteristic green fluorescence, which would not be the case if nucleic acids were not present in the crystals.

An identical excitation spectrum has been tested using Crystal-Tube Gel Tube R glass capillaries (GT-R; CFS-MB2004-CRT200). These have a thick wall of approximately 0.5 mm (Figs. 4 ▶ *a* and 4 ▶ *b*). GT-R were used to apply a variation of the counter-diffusion liquid-to-liquid crystallization approach. The capillary was filled with protein solution and a piece of gel tubing was attached to one end of the capillary (McPherson, 1999 ▶). For intrinsic fluorescence imaging, the capillaries need to be taken out of the Granada Crystallization boxes owing to the intrinsic fluorescence properties of the box itself. Crystals of mistletoe lectin I were illuminated with white light and the excitation spectra were recorded (Figs. 4 ▶ ▶ *a* and 4 ▶ *b*, respectively). Additionally, common glass capillaries with thin walls of approximately 0.1 mm have been investigated (Figs. 4 ▶ *c* and 4 ▶ *d*). With the assumption that most precipitants contain no aromatic groups, intrinsic fluorescence can distinguish between protein and precipitant crystals. Fig. 5 ▶ shows lysozyme crystals among sodium chloride crystals in the well of an MRC2 crystallization plate (MRC 96-well crystallization plate; catalog No. MD11-00U-100, Molecular Dimensions, UK), covered with sealing film (ClearVue Sheets; catalog No. MD6-01S) when illuminated with the excitation spectrum (Fig. 5 ▶ *a*) and when illuminated with white light (Fig. 5 ▶ *b*).

Intrinsic fluorescence provides the ability to identify crystals formed by a complex of a protein and a nucleic acid. Specific fluorescence dyes are available that allow one to extend intrinsic fluorescence imaging, for example for nucleic acids stained with the SYBR Gold nucleic acid gel stain (Life Technologies, catalog No. S-11494). This dye binds specifically but not covalently to nucleic acids, similar to the Izit approach (Uyeno *et al.*, 2004 ▶), and provides the polymer with a sufficient fluorescence property to apply intrinsic fluorescence imaging. An example is shown in Figs. 5 ▶(*c*), 5 ▶(*d*) and 5 ▶(*e*). Crystals of a 40-mer mirror-image, non-natural l-chirality RNA aptamer with the pro-inflammatory chemokine l-CLL2 (monocyte chemoattractant protein 1), a natural protein composed of regular l-amino acids (Oberthür *et al.*, manuscript submitted), were investigated applying the excitation spectrum. The mirror-image RNA is identified to bind l-CCL2 with high affinity, therefore its presence in the crystals was anticipated. Fig. 5 ▶(*c*) shows crystals of the complex obtained by a hanging-drop approach using a Linbro plate (Hampton Research, catalog No. HR3-110) covered with a common siliconized cover slip illuminated by bright light. Fig. 5 ▶(*d*) shows the same crystal when illuminated with the excitation spectrum. The characteristic blue fluorescence indicates the presence of protein in the crystals. Fig. 5 ▶(*e*) shows the intrinsic fluorescence imaging after the addition of SYBR Gold to the droplet to an approximately 1:1000 dilution. The droplet was again illuminated with the excitation spectrum. The characteristic green fluorescence indicates the anticipated presence of nucleic acids in the crystals.

The use of an added noncovalently binding fluorescent species follows the Izit approach to identifying protein crystals (Izit Crystal Dye, Hampton Research, Aliso Viejo, California, USA; Cosenza *et al.*, 2003 ▶). In this case the added dye is not fluorescent, or is only weakly so, until it binds to a suitable location on the protein molecule, which is typically a hydrophobic region. However, in contrast to the Izit approach the fluorescent dye is added to the protein solution prior to setting up the crystallization trials. The fluorescent dye employed was 1,8-ANS (1-anilinonapthalene-8-sulfonic acid; Invitrogen), a known sensitive indicator for protein conformational studies (Semisotnov *et al.*, 1991 ▶). A similar approach using the fluorescent probe SYBR Green has been described for identifying DNA in crystals (Kettenberger & Cramer, 2006 ▶).

Suggestions of adding a chemical as a diagnostic tool to the crystallization procedure bring about immediate concerns about the effects that this will have for obtaining crystals and on the quality of the crystals obtained, despite the predilection for adding a variety of chemicals for optimization purposes. There is evidence that the addition of 1,8-ANS does affect the structure of a protein (Schönbrunn *et al.*, 2000 ▶), where it was found to induce a structural change in the protein MurA. However, in this case the molar ratio of protein:ANS was ∼1:2 and all of the protein molecules had probe bound to them. Groves *et al.* (2007 ▶) addressed both of these issues. Using a 100 ms cutoff exposure time, 1,8-ANS was found to be suitable for crystal identification down to a concentration of 0.9 µm. Protein concentrations in the crystalline state are typically in the 1 m*M* range, resulting in the probe being at ∼0.1% of the protein concentration, which is well below that used by Schönbrunn *et al.* (2000 ▶). When tested with a range of model proteins the presence of 1,8-ANS did not affect the crystallization outcomes. The effects on diffraction resolution were tested using the protein Ppm1p with 1,8-ANS at a concentration of 9 µ*M*. No effects on the diffraction resolution could be discerned on the basis of the diffraction data.

Subsequent work has described a simple instrument for making fluorescence measurements using the added-probe approach (Watts *et al.*, 2010 ▶). Using 1,8-ANS as the fluorescent probe, the instrument uses an array of 96 light-emitting diodes with a peak emission wavelength of 365 nm for excitation, one for each position of a 96-well plate. While a transmission geometry is employed, removal of the excitation signal from the emission is greatly facilitated by the 1,8-ANS Stokes shift of ∼100 nm. The method images the entire plate in one image, and as a result suffers from a rather low resolution of 35 µm per pixel. The method has the strong benefit of being easily implementable. However, the instrument is fixed in the excitation wavelength, and to use another dye one would have to assemble a suitable array using the appropriate LEDs.

This introduces the use of LEDs as excitation sources for fluorescence applications. LEDs have many advantages over previous bulb-type sources: they emit over a narrow wavelength range, they are usually very low-power and low-cost, they have very long lifetimes, typically in the ≥50 000 h range, they are available in a wide range of peak wavelengths, making it easy to match them to specific fluorophores, and compared with lasers they are generally ‘eye safe’. One failing is the lack of spectral range for a single LED, requiring a separate source for each excitation wavelength.

The third fluorescence method is to covalently attach a fluorescent probe to the protein, taking care to remove free, nonbound, probe prior to using the protein for crystallization trials. The method is called trace fluorescent labeling (TFL), as the goal is to only label ∼0.2%, or ∼2 per thousand, of the molecules. The labeling site is left to the experimenter and the available fluorescent probes, with side-chain and N-terminal amines, thiols, hydroxyls and carboxyl groups of proteins available for reaction. The easiest groups to target are side-chain and N-terminal amines, using fluorescent probes having a reactive succinimidyl ester. The reaction forms a very stable amide bond. The reaction solution must be free of primary and secondary amines other than those on the protein. By adjusting the reaction pH, one can target either the N-terminal amine with a reaction pH of ∼7.5 or side-chain amines with a pH of 8.5–9.5. With most proteins to be crystallized this will involve several buffer exchanges, first to move the protein into a reaction buffer and then to change it back to the crystallization buffer and remove the unbound probe. As originally described, the labeling process was somewhat tedious, using gel-filtration columns to carry out the buffer exchanges (Forsythe *et al.*, 2006 ▶). However, it has now been revised to a much quicker procedure using centrifugal desalting columns that can be carried out in as little as 10 min (Pusey *et al.*, 2007 ▶; Pusey *et al.*, submitted). The major time saving derives from the avoidance of protein-concentration procedures, while limitation on the derivatization levels comes from carrying out the reaction on only a fraction of the protein to limit the final labeled population.

The sensitivity of the crystal nucleation process to the covalent labeling process was directly addressed in the initial study. Three different proteins were tested using known crystallization conditions, with the probe-labeled protein varying from 0.1 to 5% (ribonuclease) or 10% (thaumatin and lysozyme). Lysozyme was tested using both randomly labeled side-chain and N-terminally labeled protein. The results were protein-specific, with thaumatin showing some increase in the nucleation rate with increasing levels of labeled protein, and ribonuclease and lysozyme being essentially unchanged over the range. For all three proteins there was no change at labeled protein levels of ≤1%. A more recent study using a larger pool of proteins has also shown no effects of the probe at TFL levels on the screening success rate compared with unlabeled protein (Pusey *et al.*, submitted)

The effects on crystal diffraction quality were also examined, with labeling levels varying from 0 (control) to 5% for insulin, ribonuclease and N-terminally labeled lysozyme and to 10% for randomly labeled lysozyme and thaumatin. Values of *R*_merge_ were found to not change significantly over the labeling range tested for all of the data and for the highest resolution data shell. It should be pointed out that the probe used, 5,6-carboxyrhodamine, has a molecular weight of 555, which is approximately one tenth of the molecular weight of insulin (Forsythe *et al.*, 2006 ▶).

**Figure 6:**
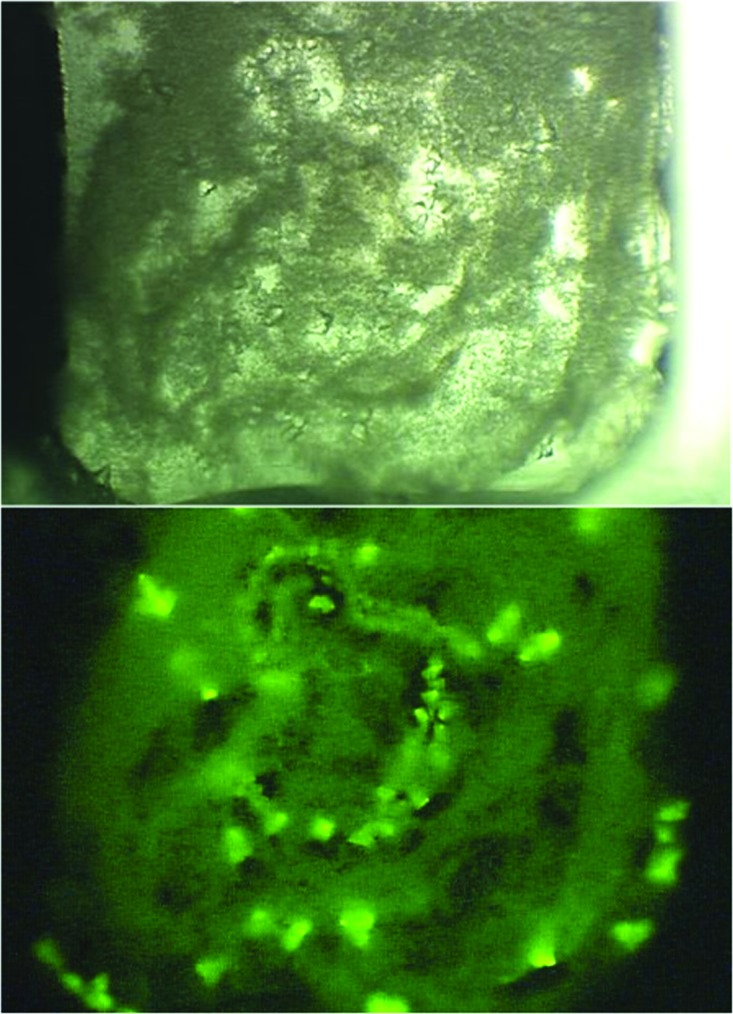
Observing crystals buried in precipitate using TFL protein. The upper panel is using white light and the lower panel is the fluorescent image.

An original impetus for the TFL approach was to distinguish salt crystals from protein crystals. However, use of the method quickly revealed additional benefits. One of the first was the ability to detect hidden or buried crystals more easily. Fig. 6 ▶ shows white-light and fluorescent images of crystals buried in precipitate. While the precipitate also fluoresces, the greater packing density of the crystals results in their greater fluorescence intensity, making them readily apparent even through buried in precipitate.

**Figure 7:**
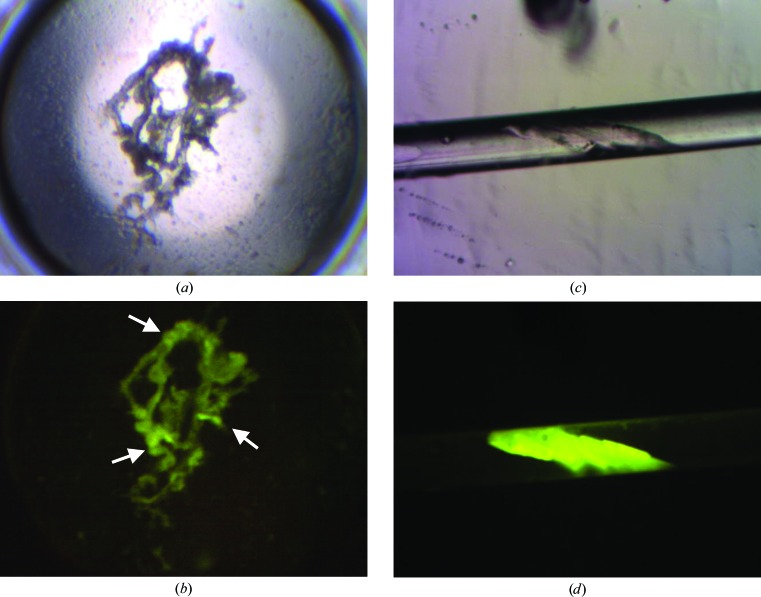
Conversion of a ‘bright spot’ outcome for a TFL protein to crystallization conditions. (*a*) shows the white-light image of the droplet, while (*b*) is the corresponding fluorescence image. The arrows in (*b*) indicate ‘bright spots’, which are taken to indicate that these are potential lead crystallization conditions. (*c*) and (*d*) are the corresponding white-light and fluorescence images of the optimized conditions, with optimization carried out using capillary counter-diffusion. The capillary internal diameter is 0.3 mm.

Perhaps the most surprising finding was that of ‘bright spots’ (Pusey *et al.*, 2007 ▶; Pusey *et al.*, submitted). Early in the method-development process it was noted that there were wells with precipitate which had noticeably brighter regions in them, often with intensities similar to that for crystals. Close observation at higher magnification did not show any distinguishable structure corresponding to these bright regions. Furthermore, the small size, generally <15–20 µm, generally low occurrence and typical location within precipitate of these spots did not favor their extraction for X-ray analysis. Intensity is a function of the packing density, and crystals are typically the most densely packed form for the protein. This higher intensity thus suggests that there is some locally ordered structure of the protein molecules, and we concluded that these may indicate an initial crystallization process and thus potential crystallization conditions. As shown in Fig. 7 ▶, these conclusions were often verified upon subsequent optimization screening around these conditions. These are lead conditions that would not be evident under white-light viewing only, and represent a significant increase in the number of found ‘hits’. A more extended study has shown that ∼33% of ‘bright spot’ outcomes are converted to crystals upon optimization (Pusey *et al.*, submitted).

**Figure 8:**
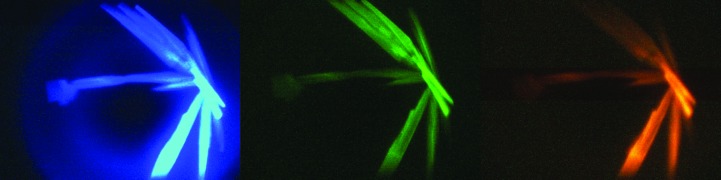
Proof of concept of using multicolor TFL for crystallization of complexes. From left to right the fluorescing species are Pacific Blue, carboxyrhodamine and Cascade Yellow.

The advantage of the TFL approach is that one can control the fluorescence wavelength employed to avoid potentially interfering compounds or to better match existing equipment. Another advantage is that one can use multiple colors, making this a very useful technique when working with complexes. As a proof of concept, aliquots of a hyperthermophile-derived inorganic pyrophosphatase were separately trace fluorescently labeled with the fluorescent probes Cascade Yellow (excitation at 405 nm, emission at 550–570 nm), carboxyrhodamine (excitation at 525 nm, emission at 540–560 nm) and Pacific Blue (excitation at 405 nm, emission at 450–460 nm). The labeled aliquots were mixed together and the resulting protein solution was set up in a crystallization screening trial. Fig. 8 ▶ shows the results. The microscopy system was set up such that each probe species was individually excited and the resulting emission imaged. Cascade Yellow and Pacific Blue are both excited at 405 nm, but can be individually imaged by changing the emission filter.

The above review covers fluorescent approaches to screening that are readily available to the home laboratory, either commercially or through a ‘home-brew’ approach. Once the principles of the process are understood, it is a relatively simple matter to find a means of implementing them. Indeed, the intrinsic fluorescence approach is designed to be readily adaptable to laboratory microscopy and/or plate imaging systems, while the added-probe and TFL approaches can make use of in-house fluorescence microscopes. The advantages of automated systems is that one does not have to manually review results well by well, but can rapidly survey the results using an array of stored thumbnail images.

A fourth approach to fluorescence, which is not covered above, is not as easily implementable. This approach, two-photon fluorescence, has been demonstrated for protein crystallization screening applications (Madden *et al.*, 2011 ▶). The method is based upon two photons of light at half the energy per photon, and therefore twice the wavelength, being absorbed by the fluorescing molecule. For 280 nm intrinsic fluorescence this would be light at 560 nm, although the demonstrated system used 515 nm with a reduction in the excitation. The two photons must be absorbed by the fluor within 10^−15^–10^−16^ s (Xu & Webb, 1997 ▶), necessitating a very high photon flux. As a result, fluorescence only occurs at the focal point of the imaging objective, as shown at http://www.sfb596.med.uni-muenchen.de/news/archive/2009/20090803/index.html. The small spot of two-photon fluorescence requires that scanning of the area to be imaged needs to take place in two dimensions if just a planar area is to be imaged and in three dimensions if a volume is to be imaged. The requirement for high photon flux and thus a higher powered pulsed laser source to provide the photon flux and scanning to build each image are limitations on the ready accessibility of this approach to the smaller laboratory. However, it does enable the use of intrinsic fluorescence while bypassing the UV transmissive optical requirements, and the method bears watching for future developments.

Overall, fluorescence, whether intrinsic or by using TFL, can be a powerful aid in macromolecule crystallization. Here, we have only discussed its use in screening for crystals, although other applications in the field of macromolecule crystallization and crystal growth are possible. Simple instrumentation incorporating the requisite basic functionality for the three main approaches discussed can be realised in even a small structural biology laboratory. The benefits obtained are powerful aids in interpreting the screening results as well as obtaining potential insights leading to additional, previously unrealized, lead conditions.
